# Retrospective analysis of the effectiveness and tolerability of nab‐paclitaxel in Chinese elderly patients with advanced non‐small‐cell lung carcinoma

**DOI:** 10.1111/1759-7714.13356

**Published:** 2020-03-11

**Authors:** Shuhang Wang, Qiuping Liang, Yujia Chi, Minglei Zhuo, Tongtong An, Jianchun Duan, Zhijie Wang, Yuyan Wang, Jia Zhong, Xue Yang, Hanxiao Chen, Jie Wang, Jun Zhao

**Affiliations:** ^1^ Clinical Cancer Center, National Cancer Center/National Clinical Research Center for Cancer/Cancer Hospital Chinese Academy of Medical Sciences and Peking Union Medical College Beijing China; ^2^ Department of Respiratory and Critical Care The Third People's Hospital in Chengdu Chengdu China; ^3^ Department of Thoracic Medical Oncology‐I, Key Laboratory of Carcinogenesis and Translational Research (Ministry of Education), Peking University Cancer Hospital & Institute, Beijing China; ^4^ Department of Medical Oncology, National Cancer Center/National Clinical Research Center for Cancer/Cancer Hospital Chinese Academy of Medical Sciences and Peking Union Medical College Beijing China

**Keywords:** Albumin‐bound paclitaxel, elderly, non‐small‐cell lung cancer

## Abstract

**Background:**

Previous trials have suggested that elderly patients with non‐small‐cell lung cancer (NSCLC) could benefit from nanoparticle albumin‐bound paclitaxel (nab‐paclitaxel). Real‐world data on the elderly Chinese population are lacking. This study aimed to analyze the effectiveness and tolerability of nab‐paclitaxel in Chinese elderly patients (≥65 years) with advanced NSCLC.

**Methods:**

This study included 76 patients with a primary diagnosis of IIIB‐IV NSCLC from January 2010 to December 2017 at Peking University Cancer Hospital, who received nab‐paclitaxel (125 or 130 mg/m^2^ i.v.) every three weeks. The overall survival (OS), progression‐free survival (PFS), objective response rate (ORR), disease control rate (DCR), and adverse events (AEs) were analyzed.

**Results:**

There were 12 patients who received nab‐paclitaxel as the first‐line treatment (seven also received carboplatin), and 64 received nab‐paclitaxel as the latter‐line treatment. The overall ORR, DCR, median PFS, and median OS were 14.5%, 69.7%, 5.2 months, and 12.2 months, respectively. The Eastern Cooperative Oncology Group performance status of one and the age of 70–74 years were independently associated with longer OS, while early treatment line of nab‐paclitaxel and age of 70–74 years were independently associated with longer PFS. The most common AEs were anemia, leukopenia, gastrointestinal reaction, fatigue, and peripheral neuropathy, which were all manageable. Dose adjustment or treatment discontinuation was encountered in 10 patients because of AEs. The incidence of AEs was not different among age subgroups.

**Conclusions:**

Nab‐paclitaxel has a good clinical response profile in Chinese elderly patients with stage IIIB–IV NSCLC. Prospective clinical trials are needed to confirm these results.

**Key points:**

**Significant findings of the study**

Nanoparticle albumin‐bound paclitaxel (nab‐paclitaxel) has a good clinical response profile in Chinese elderly (≥65 years) patients with stage IIIB‐IV non‐small‐cell lung cancer (NSCLC), with acceptable and manageable adverse events.

**What this study adds**

Preliminary evidence shows a good clinical response from treatment with nab‐paclitaxel in Chinese elderly patients with advanced NSCLC.

## Introduction

Non‐small‐cell lung cancers (NSCLCs) are the most frequent (85%–90%) cause of malignant lung tumors, usually affecting adults who smoke and are aged ≥65 years.^1^, ^2^ In China, the annual incidence of lung cancer is approximately 36.7 per 100 000 person‐years, with a mortality of 28.5 per 100 000 person‐years.^3^

The incidence and prevalence of NSCLC have decreased in younger patients and increased or reached a plateau in elderly patients, highlighting the need for suitable treatment in the elderly subgroup.^4^, ^5^ Older patients often present with poor performance status and/or comorbidities,^6^ and many receive only the best supportive care.^2^, ^6^ Elderly patients are often underrepresented in clinical trials because of anticipated toxic effects, frailty, and comorbidities, and new therapeutic options are limited.^7^, ^8^ The median age in large clinical trials is 60.9 years,^9^ whereas the median age for all patients diagnosed with NSCLC is 70 years.^10^ As NSCLC occurs mostly after 70 years of age, more effective and better‐tolerated therapeutic options for the elderly are needed.

Nanoparticle albumin‐bound paclitaxel (nab‐paclitaxel), with a mean particle size of 130 nm, was developed to improve the therapeutic effect of paclitaxel. In the phase III trial CA031, weekly nab‐paclitaxel plus every three‐week (q3w) carboplatin (nab‐P/C) versus standard q3w solvent‐based paclitaxel (sb‐paclitaxel) plus carboplatin (sb‐P/C) led to a significantly higher objective response rate (ORR; primary endpoint; 33% vs. 25%, *P* = 0.005), a one‐month increase in median overall survival (OS; median, 12.1 vs. 11.2 months, *P* = 0.271), and an improved safety profile in the first‐line treatment of patients with advanced NSCLC.^11^ In subgroup analyses, ORR was improved in patients with squamous histological features (41% vs. 24%, *P* < 0.001), and OS was significantly improved (median, 19.9 vs. 10.4 months, *P* = 0.009) in patients ≥70 years or ≥60 years of age (median, 13.8 vs. 11.0 months, *P* = 0.009). In the phase II trial ABOUND.70+, weekly nab‐P/C, either continuously or with a one‐week break, in elderly patients with advanced NSCLC was well tolerated and effective, with ORR of 24% and 40% and median OS of 15.2 months and 16.2 months, respectively.^12^

Therefore, previous trials suggested that elderly patients with NSCLC could benefit from nab‐paclitaxel, but real‐world data on the elderly Chinese population are lacking. The present study aimed to analyze the effectiveness and tolerability of nab‐paclitaxel in Chinese elderly patients (≥65 years^13^) with stage IIIB–IV NSCLC.

## Methods

### Study design and patients

This retrospective study included patients who received a primary diagnosis of advanced NSCLC at Peking University Cancer Hospital between January 2010 and December 2017. The study was approved by the ethics committee of Peking University Cancer Hospital. The need for individual consent was waived by the committee because of the retrospective nature of the study.

The inclusion criteria were as follows: (i) pathological diagnosis of NSCLC; (ii) stage IIIB–IV; and (iii) ≥1 cycle of nab‐paclitaxel treatment. Patients with incomplete demographic data or missing histologic types were excluded.

### Treatment

Patients received nab‐paclitaxel (Abraxane; Celgene, Summit, NJ, USA) 125 or 130 mg/m^2^ i.v. on days 1 and 8 every three weeks.^14^, ^15^, ^16^ The dose could be adjusted by 10% to 20% according to the patient's condition and the experience of the physician. The number of treatment cycles was commonly 4–6 cycles, which could be adjusted according to effectiveness and tolerability.

### Response evaluation

Treatment response was evaluated every two cycles using chest computed tomography according to the Response Evaluation Criteria in Solid Tumors (RECIST) 1.1. The treatment response was reviewed by two experienced radiologists who were blinded to patient information. The ORR included the complete response (CR) and partial response (PR) rates. The disease control rate (DCR) included the CR, PR, and stable disease (SD) rates. The progression‐free survival (PFS) was defined as the time from the initiation of nab‐paclitaxel chemotherapy to disease progression or death, whichever came first. The OS was defined as the time from the initiation of nab‐paclitaxel chemotherapy to death. Adverse events (AEs) were graded according to the Common Terminology Criteria for Adverse Events 4.0.

### Statistical analysis

Statistical analyses were carried out using SPSS 16.0 (SPSS Inc., IL, USA). Categorical data were presented as frequency (percentage) and analyzed using the chi‐square test or Fisher exact test, as appropriate. PFS and OS were estimated using the Kaplan‐Meier method, and the log‐rank test was used to compare the survival curves. The univariable Cox proportional hazards regression model was used to examine the potential associated factors. Proportional hazards tests based on Grambsch & Therneau^17^ were used to ensure the appropriateness of the model. Factors with *P*‐values less than 0.1 were entered into a multivariable Cox proportional hazard regression model (backward method). A two‐sided *P*‐value <0.05 was considered statistically significant. As all analyses were not predefined, and the study was not powered for any hypothesis testing, the *P*‐values were only for display purposes.

## Results

### Patients and treatment exposure

Between January 2010 and December 2017, a total of 239 patients with stage IIIB–IV NSCLC were treated with nab‐paclitaxel. Of these, 153 patients were excluded due to being age < 65 years, and 10 patients were excluded due to incomplete data. Finally, 76 patients were included (52 male [68.4%] and 24 female [31.6%]). The demographic and pathological characteristics of the 76 patients are presented in Table 1. Most patients had a good Eastern Cooperative Oncology Group (ECOG) performance status, but seven patients had an ECOG performance status of two (9.2%). The median age was 72 years (range 65–82); 39.5% (*n* = 30) of the patients were 65–69 years old, 28.9% (*n* = 22) were 70–74 years old, and 31.6% (*n* = 24) were ≥75 years old. A total of 41 patients (53.9%) had squamous cell carcinoma (SCC), and 35 patients (46.1%) had nonsquamous cell carcinoma (non‐SCC), including 32 with adenocarcinoma and three with not‐otherwise‐specified (NOS) lung cancer. There were 15 patients (19.7%) who harbored epidermal growth factor receptor (*EGFR*) mutations. Nearly half of the patients had cardiovascular diseases (*n* = 36, 47.4%). Most patients were heavily treated; only 12 patients (15.8%) received nab‐paclitaxel as the first‐line treatment (seven also received carboplatin), 16 patients (21.1%) received nab‐paclitaxel as the second‐line treatment, and 48 patients (63.2%) received nab‐paclitaxel as the third‐ or further‐line treatment. A total of 40 patients had previously received taxane treatment. A total of 67 patients (88.2%) received nab‐paclitaxel monotherapy, while nine patients received nab‐paclitaxel combined with platinum or bevacizumab. Among the 15 patients with *EGFR* mutation, one patient (6.7%) received second‐line nab‐paclitaxel, and 14 patients (93.3%) received third‐ or further‐line nab‐paclitaxel. Among the 61 patients without *EGFR* mutation, 12 (19.7%) received first‐line nab‐paclitaxel, 15 (24.6%) received second‐line nab‐paclitaxel, and 34 (55.7%) received third‐ or further‐line nab‐paclitaxel. The median number of treatment cycles of nab‐paclitaxel was two.

**Table 1:** Baseline characteristics of the patients

Characteristics	Patients (*n* = 76)
Age (years), *n* (%)	
65–69	30 (39.5)
70–74	22 (28.9)
≥75	24 (31.6)
Male, *n* (%)	52 (68.4)
ECOG performance status, *n* (%)	
0	22 (28.9)
1	47 (61.8)
2	7 (9.2)
Smoking (pack‐year), *n* (%)	
>40	19 (25.0)
≤40	57 (75.0)
Stage, *n* (%)	
III	20 (26.3)
IV	56 (73.7)
Histology, *n* (%)	
Nonsquamous cell carcinoma	35 (46.1)
Squamous cell carcinoma	41 (53.9)
EGFR status, *n* (%)	
Wild‐type	61 (80.3)
Mutation	15 (19.7)
Baseline cardiovascular disease, *n* (%)	36 (47.4)
Treatment line of nab‐paclitaxel, *n* (%)	
First‐line	12 (15.8)
Second‐line	16 (21.1)
Third‐line or further	48 (63.2)
Prior taxane, *n* (%)	40 (52.6)
Current treatment, *n* (%)	
Nab‐paclitaxel monotherapy	67 (88.2)
Nab‐paclitaxel + platinum	7 (9.2)
Nab‐paclitaxel + bevacizumab	2 (2.6)

ECOG, Eastern Cooperative Oncology Group; EGFR, epidermal growth factor receptor.

### Treatment response

Of the 76 patients, the data of 71 patients were available for efficacy evaluation. The tumor response of the patients is summarized in Table 2. No CR was observed, whereas 11 patients had PR. The ORR was 14.5%. A total of 42 patients had SD. The DCR was 69.7%, and 18 patients had progressive disease (23.7%).

**Table 2:** Treatment response

Response, *n* (%)	Patients (*n* = 76)
Partial response	11 (14.5)
Stable disease	42 (55.3)
Progressive disease	18 (23.7)
Not evaluated	5 (6.6)
Objective response rate	11 (14.5)
Disease control rate	53 (69.7)

### Factors associated with treatment response

**Figure 1:**
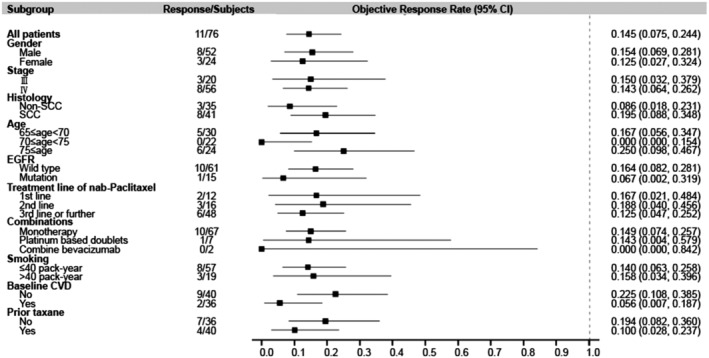
Forest plot of the objective response rate. CI, Confidence interval; CVD, cardiovascular disease; EGFR, epidermal growth factor receptor; SCC, squamous cell carcinoma.

**Figure 2:**
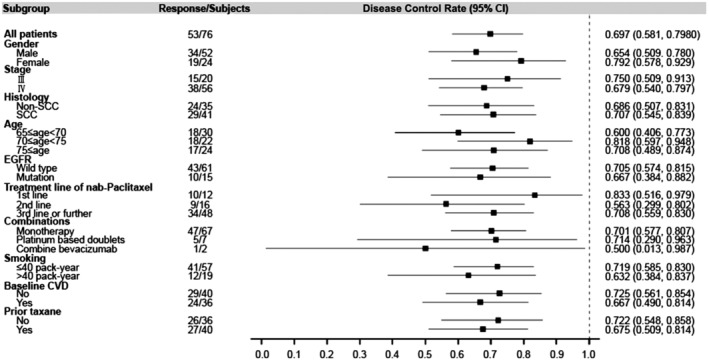
Forest plot of the disease control rate. CI, Confidence interval; CVD, cardiovascular disease; EGFR, epidermal growth factor receptor; SCC, squamous cell carcinoma.

The associations of patient demographics or clinical characteristics with ORR and DCR to nab‐paclitaxel were analyzed (Table 3). Regarding the ORR, age groups were associated with ORR (*P* = 0.034). No factor was associated with DCR. Figures 1 and 2 present the forest plots of the factors associated with ORR and DCR.

**Table 3:** Treatment response in different subgroups

Characteristics	ORR	*P*‐value	DCR	*P*‐value
Age (years), *n* (%)		0.034		0.263
65–69 (*n* = 30)	5 (16.7)		18 (60.0)	
70–74 (*n* = 22)	0		18 (81.8)	
≥75 (*n* = 24)	6 (25.0)		17 (70.8)	
Sex, *n* (%)		>0.999		0.288
Male (*n* = 52)	8 (15.4)		34 (65.4)	
Female (*n* = 24)	3 (12.5)		19 (79.2)	
Smoking (pack‐year), *n* (%)		>0.999		0.566
>40 (*n* = 19)	3 (15.8)		12 (63.2)	
≤40 (*n* = 57)	8 (14.0)		41 (71.9)	
Stage, *n* (%)		>0.999		0.777
III (*n* = 20)	3 (15.0)		15 (75.0)	
IV (*n* = 56)	8 (14.3)		38 (67.9)	
Histology, *n* (%)		0.208		>0.999
Nonsquamous cell carcinoma (*n* = 35)	3 (8.6)		24 (68.6)	
Squamous cell carcinoma (*n* = 41)	8 (19.5)		29 (70.7)	
EGFR status, *n* (%)		0.682		0.762
Wild‐type (*n* = 61)	10 (16.4)		43 (70.5)	
Mutation (*n* = 15)	1 (6.7)		10 (66.7)	
Treatment line of nab‐paclitaxel, *n* (%)		0.717		0.317
First‐line (*n* = 12)	2 (16.7)		10 (83.3)	
Second‐line (*n* = 16)	3 (18.8)		9 (56.3)	
Third‐line or further (*n* = 48)	6 (12.5)		34 (70.8)	
Prior taxane, *n* (%)		0.332		0.803
Yes (*n* = 40)	4 (10.0)		27 (67.5)	
No (*n* = 36)	7 (19.4)		26 (72.2)	
Current treatment, *n* (%)		>0.999		0.840
Nab‐paclitaxel monotherapy (*n* = 67)	10 (14.9)		47 (70.1)	
Nab‐paclitaxel + platinum (*n* = 7)	1 (14.3)		5 (71.4)	
Nab‐paclitaxel + bevacizumab (*n* = 2)	0		1 (50.0)	

DCR, disease control rate; EGFR, epidermal growth factor receptor; ORR, objective response rate.

### Survival

**Figure 3:**
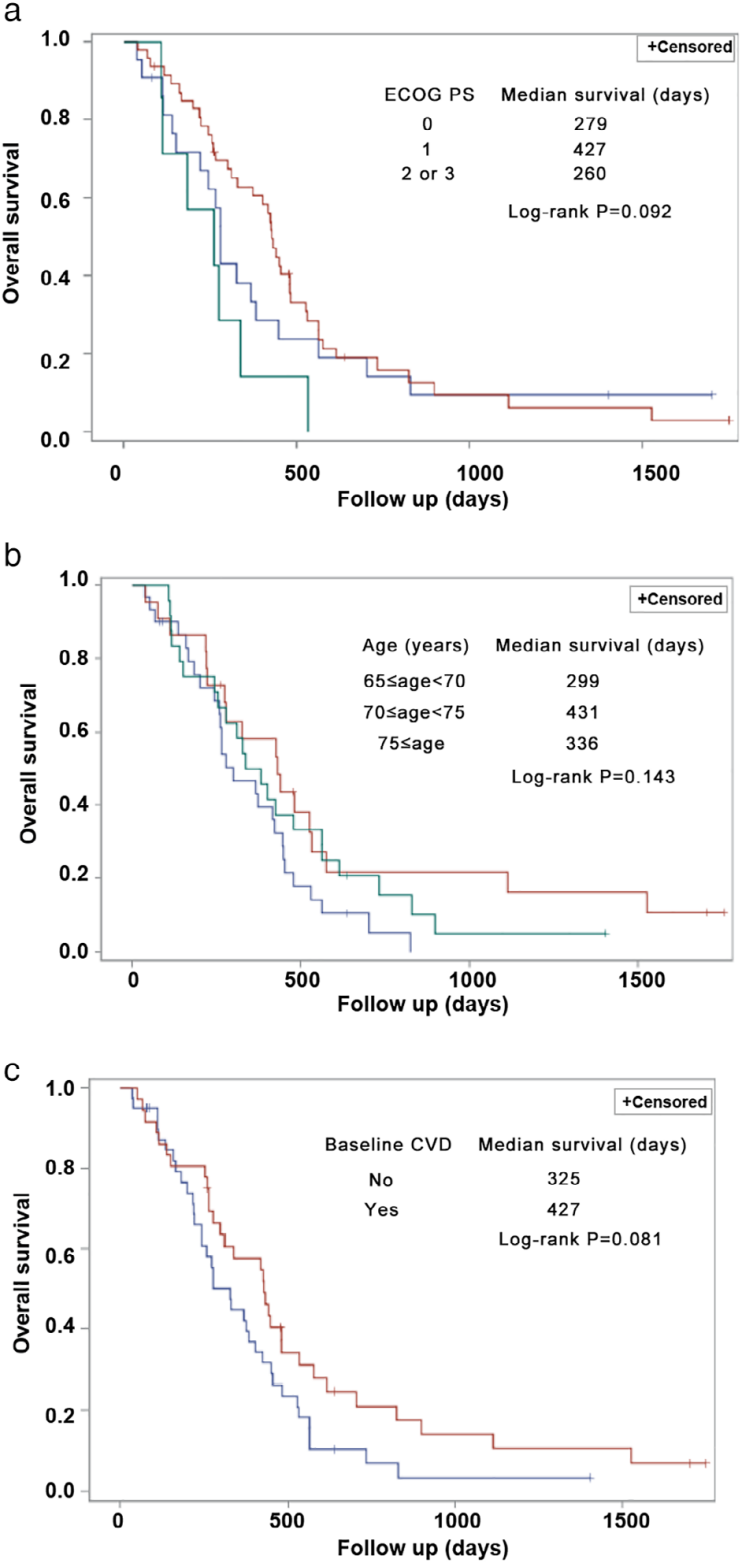
Overall survival in subgroups. (**a**) Eastern Cooperative Oncology Group performance status (ECOG PS). ECOG PS (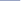) 0, (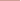) 1 and (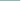) 2 or 3. (**b**) Age. Age (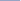) 65 ≤ age < 70, (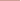) 70 ≤ age < 75 and (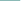) 75 ≤ age. (**c**) Baseline cardiovascular disease (CVD). Baseline CVD (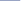) no and (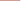) yes.

The median PFS was 5.2 months (95% confidence interval [CI], 4.4–6.0), and the median OS was 12.2 months (95% CI, 7.2–17.2). The PFS/OS in the patient subgroup with different demographic or clinical characteristics was also analyzed. The univariable Cox proportional hazard regression models showed that ECOG performance status of 1 (vs. 2), age of 70–74 years (vs. 65–69), and the presence of cardiovascular diseases were potential prognostic factors for longer OS (*P* < 0.1) (Fig 3 and Table 4). Therefore, a multivariable Cox proportional hazard regression model was used. The findings revealed that both ECOG performance status of 1 (vs. 2; hazard ratio [HR] = 0.359, 95% CI, 0.156–0.827, *P* = 0.016) and age of 70–74 years (vs. 65–69; HR = 0.499, 95% CI, 0.263–0.947, *P* = 0.034) were independently associated with longer OS (Table 4).

**Table 4:** Cox regression analysis for overall survival

	Univariable		Multivariable	
Characteristics	HR (95% CI)	*P*‐value	HR (95% CI)	*P*‐value
Age (years) (vs. 65–69)				
70–74	0.544 (0.291–1.017)	0.056	0.499 (0.263–0.947)	0.034
≥75	0.709 (0.400–1.255)	0.238	0.697 (0.393–1.236)	0.217
Sex (female vs. male)	0.790 (0.470–1.329)	0.375	−	−
ECOG performance status (vs. 2)	−	−	−	−
0	0.510 (0.211–1.234)	0.135	0.410 (0.164–1.022)	0.056
1	0.415 (0.184–0.941)	0.035	0.359 (0.156–0.827)	0.016
Smoking (pack‐year) (≤40 vs. >40)	0.768 (0.439–1.343)	0.354	−	−
Stage (IV vs. III)	1.606 (0.890–2.897)	0.116	−	−
Histology (non‐SCC vs. SCC)	0.936 (0.577–1.518)	0.790	−	−
EGFR status (mutation vs. wild‐type)	1.032 (0.579–1.838)	0.915	−	−
Baseline cardiovascular disease (yes vs. no)	0.648 (0.396–1.059)	0.084	−	−
Treatment line of nab‐paclitaxel (vs. first‐line)	−	−	−	−
Second‐line	1.444 (0.640–3.259)	0.377	−	−
Third‐line or further	1.128 (0.564–2.258)	0.733	−	−
Prior taxane (yes vs. no)	0.865 (0.533–1.405)	0.558	−	−
Current treatment (vs. nab‐paclitaxel monotherapy)	−		−	−
Nab‐paclitaxel + platinum	0.482 (0.175–1.330)	0.159	−	−
Nab‐paclitaxel + bevacizumab	2.529 (0.603–10.613)	0.205	−	−

CI, confidence interval; ECOG, Eastern Cooperative Oncology Group; EGFR, epidermal growth factor receptor; HR, hazard ratio; SCC, squamous cell carcinoma.

**Figure 4:**
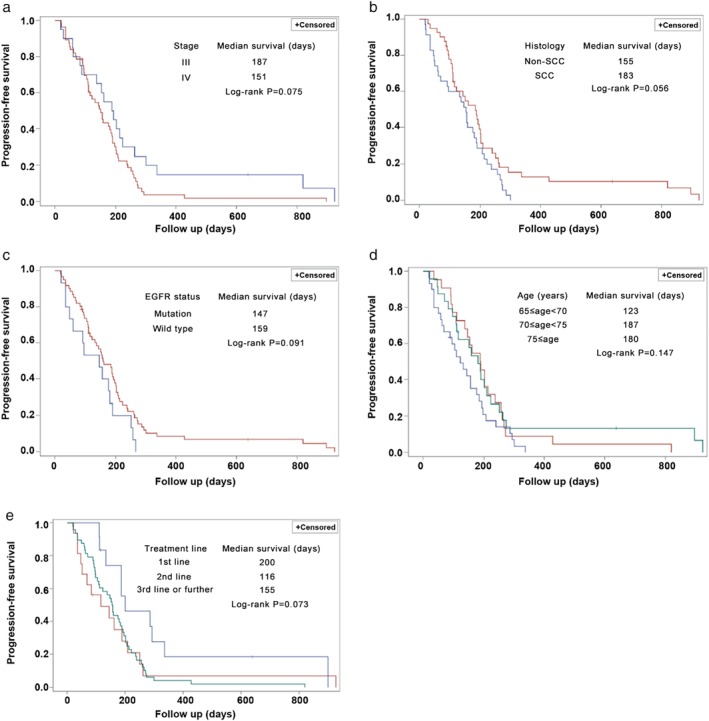
Progression‐free survival in subgroups. (**a**) Stage. Stage (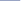) III and (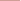) IV. (**b**) Histology. Histology (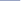) non‐SCC and (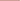) SCC. (**c**) *EGFR* status. EGFR status (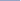) mutation and (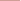) wild‐type. (**d**) Age. Age (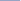) 65 ≤ age < 70, (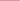) 70 ≤ age < 75 and (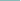) 75 ≤ age. (**e**) Treatment line of nab‐paclitaxel. Treatment line (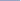) first‐line, (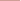) second‐line and (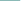) third‐ line or further. EGFR, Epidermal growth factor receptor; SCC, squamous cell carcinoma.

Regarding PFS, stage III (vs. IV), histology of SCC (vs. non‐SCC), *EGFR* wild‐type (vs. mutation), age of ≥75 years (vs. 65–69), and early use of nab‐paclitaxel (first‐line vs. second‐line, first‐line vs. third‐ or further‐line) were associated with longer PFS (*P* < 0.1) (Fig 4 and Table 5). The multivariable Cox proportional hazards regression model showed that early treatment line of nab‐paclitaxel (second‐line vs. first‐line: HR = 3.088, 95% CI, 1.282–7.440, *P* = 0.012, third‐ or further‐line vs. first‐line: HR = 2.532, 95% CI, 1.204–5.324, *P* = 0.014) and age of ≥70–74 years (vs. 65–69; HR = 0.531, 95% CI, 0.294–0.960, *P* = 0.036) were independently associated with longer PFS (Table 5).

**Table 5:** Cox regression analysis for progression‐free survival

	Univariable		Multivariable	
Characteristics	HR (95% CI)	*P*‐value	HR (95% CI)	*P*‐value
Age (years) (vs. 65–69)				
70–74	0.672 (0.384–1.176)	0.164	0.531 (0.294–0.960)	0.036
≥75	0.586 (0.329–1.045)	0.070	0.537 (0.282–1.022)	0.058
Sex (female vs. male)	0.876 (0.530–1.449)	0.607	−	−
ECOG performance status (vs. 2)	−	−	−	−
0	0.645 (0.267–1.554)	0.328	−	−
1	0.540 (0.239–1.223)	0.140	−	−
Smoking (pack‐year) (≤40 vs. >40)	0.848 (0.490–1.469)	0.557	−	−
Stage (IV vs. III)	1.635 (0.946–2.827)	0.078	1.753 (0.991–3.101)	0.054
Histology (non‐SCC vs. SCC)	1.588 (0.982–2.567)	0.059	−	−
EGFR status (mutation vs. wild‐type)	1.641 (0.916–2.937)	0.096	−	−
Baseline cardiovascular disease (yes vs. no)	0.816 (0.511–1.305)	0.397	−	−
Treatment line of nab‐paclitaxel (vs. first‐line)	−	−	−	−
Second‐line	2.135 (0.939–4.852)	0.070	3.088 (1.282–7.440)	0.012
Third‐line or further	2.179 (1.086–4.372)	0.028	2.532 (1.204–5.324)	0.014
Prior taxane (yes vs. no)	1.246 (0.777–2.000)	0.362	−	−
Current treatment (vs. nab‐paclitaxel monotherapy)	−	−	−	−
Nab‐paclitaxel + platinum	0.506 (0.216–1.187)	0.118	−	−
Nab‐paclitaxel + bevacizumab	0.731 (0.176–3.040)	0.666	−	−

CI, confidence interval; ECOG, Eastern Cooperative Oncology Group; EGFR, epidermal growth factor receptor; HR, hazard ratio; SCC, squamous cell carcinoma.

### Adverse events

The most common AEs were anemia (40.8%, *n* = 31), leukopenia (36.9%, *n* = 28), gastrointestinal reaction (23.7%, *n* = 18), fatigue (18.4%, *n* = 14), and peripheral neuritis (17.1%, *n* = 13), which were all manageable (Table 6). The incidence of grade 3 AEs was 14.5% (11/76). No grade 4 AEs were found. No AE‐related death was reported. A total of 10 patients had dose adjustment or discontinuation due to AEs. The incidence of all grade 3–4 AEs, leukopenia (all grades), anemia (all grades), and peripheral neuritis (all grades) were not different among the age subgroups (all *P* > 0.05) (Table 7). The incidence of AE‐related dose adjustment or discontinuation was similar across the age subgroups.

**Table 6:** Adverse events

	Patients (*n* = 76)			
Event, *n* (%)	Any grade	Grade 1	Grade 2	Grade 3
Anemia	31 (40.8)	23 (30.3)	8 (10.5)	0
Leukopenia	28 (36.8)	11 (14.5)	10 (13.2)	7 (9.2)
Gastrointestinal reaction	18 (23.7)	12 (15.8)	6 (7.9)	0
Fatigue	14 (18.4)	6 (7.9)	6 (7.9)	2 (2.6)
Peripheral neuropathy	13 (17.1)	8 (10.5)	4 (5.3)	1 (1.3)
Alopecia	7 (9.2)	6 (7.9)	1 (1.3)	0
ALT/AST elevation	5 (6.6)	4 (5.3)	1 (1.3)	0
Thrombocytopenia	1 (1.3)	0	0	1 (1.3)

ALT, alanine aminotransferase; AST, aspartate aminotransferase.

**Table 7:** Age (years) subgroup analysis of adverse events

Event	65–69 (*n* = 30)	70–74 (*n* = 22)	≥75 (*n* = 24)	*P‐value*
Grade 3–4 adverse events	5 (16.7)	1 (4.5)	4 (16.7)	0.399
Leukopenia (any grade)	12 (40.0)	6 (27.3)	10 (41.7)	0.552
Anemia (any grade)	9 (30.0)	12 (54.5)	10 (41.7)	0.207
Peripheral neuropathy (any grade)	3 (10.0)	7 (31.8)	3 (12.5)	0.136
Dose adjustment or treatment discontinuation due to adverse events	5 (16.7)	1 (4.5)	4 (16.7)	0.399

## Discussion

Elderly patients are often excluded from clinical trials because of their comorbidities and frailty,^7^, ^8^ but they represent the majority of the patients with NSCLC.^10^ Indeed, the median age at NSCLC diagnosis was 70 years.^10^ Moreover, a decrease in the incidence of NSCLC in the younger age groups and an increase or plateau in the elderly ones was observed.^4^, ^5^ Therefore, evidence about the clinical benefits of treatment regimens in elderly patients with NSCLC is needed. Previous trials suggested that elderly patients with NSCLC could benefit from nab‐paclitaxel, but real‐world data regarding the elderly Chinese population are lacking.

Therefore, the present study aimed to retrospectively analyze the effectiveness and tolerability of nab‐paclitaxel in Chinese elderly patients (≥65 years) with stage IIIB–IV NSCLC. The results suggested that nab‐paclitaxel had a good clinical response profile in Chinese elderly patients with stage IIIB–IV NSCLC. The complications were tolerable and manageable. Patients of ≥70 years of age might benefit more from nab‐paclitaxel compared with younger patients. Prospective clinical trials are needed to confirm these results.

Conventional sb‐taxanes (such as paclitaxel and docetaxel) destabilize the microtubules and hence prevent cell proliferation, and are among the most widely used chemotherapies.^18^, ^19^ Because taxanes are poorly soluble in water‐based solutions, polyethylated castor oil and ethanol are needed in sub‐taxane preparations to increase the taxanes' solubility.^20^, ^21^ Therefore, premedication and long infusion times are necessary to avoid hypersensitivity reactions.^20^, ^21^ In addition, the paclitaxel is trapped in micelles by the cremophor excipient, resulting in nonlinear pharmacokinetics.^22^, ^23^ Nab‐paclitaxel has been designed to avoid the solvents causing the hypersensitivity reactions.^24^ In addition, nab‐paclitaxel achieves a higher delivery rate of paclitaxel to the tumor cells.^25^ Nab‐paclitaxel is generally recognized as a relatively mild chemotherapeutic agent with good tolerability compared with other chemotherapy regimens in various solid tumors, including NSCLC.^16^, ^26^, ^27^, ^28^, ^29^, ^30^ Clinical trials showed that nab‐paclitaxel had better efficacy and safety than sb‐paclitaxel.^11^, ^31^

Data about nab‐paclitaxel for the management of elderly patients with NSCLC are rare. The CA031 clinical trial showed that patients ≥70 or ≥60 years of age had better outcomes with nab‐paclitaxel compared with younger patients.^11^ The CA031 trial also showed that nab‐paclitaxel combined with carboplatin had better outcomes than sb‐paclitaxel combined with carboplatin in elderly patients.^32^ The ABOUND.70+ was especially designed to examine the effect of nab‐paclitaxel (continuously vs. one‐week break) in elderly patients with NSCLC, and the results showed favorable outcomes.^12^ A retrospective analysis of the NCT00540514 trial showed that nab‐paclitaxel combined with carboplatin had better ORR (46% vs. 20%), OS (20 vs. nine months) and tolerability compared with sb‐P/C in elderly patients with NSCLC.^33^ Zarogoulidis *et al*.^34^ showed that first‐line nab‐paclitaxel monotherapy had better outcomes compared with best supportive care in patients ≥75 years of age with NSCLC. In the present study, the ORR was 14.5%, and DCR was 69.7%. The median PFS was 5.2 months (95% CI, 4.4–6.0), and the median OS was 12.2 months (95% CI, 7.2–17.2). The most common AEs were anemia (40.8%), leukopenia (36.9%), gastrointestinal reaction (23.7%), fatigue (18.4%), and peripheral neuritis (17.1%), which were all manageable. The incidence of grade 3 AEs was 14.5%. No grade 4 AEs or deaths were found. This was generally comparable to the trials of nab‐paclitaxel in elderly patients with NSCLC.

The multivariable analyses showed that ECOG performance status of 1 (vs. 2) and age of 70–74 years (vs. 65–69) were independently associated with longer OS, while early treatment line of nab‐paclitaxel (first‐line vs. second‐line, first‐line vs. third‐ or further‐line) and age of 70–74 years (vs. 65–69) were independently associated with longer PFS. Sugiyama *et al*.^31^ identified the performance status and high lactate dehydrogenase levels as the independent predictors of poor OS in patients with refractory/relapsed NSCLC treated with nab‐paclitaxel. A nationwide (US) study showed that first‐line nab‐paclitaxel was associated with better OS compared with later‐line treatment in patients with lung SCC.^35^ These studies generally supported the results of the present study. Additional studies are required to identify those patients with NSCLC who can benefit the most from nab‐paclitaxel.

Elderly patients may have tumors with a lower degree of malignancy and may receive higher levels of care in the real world because of the various comorbidities. Hence, various factors may result in longer survival. The multivariable regression analysis showed that the OS and PFS of the patients in the 70–74 age group were significantly higher than those in the 65–69 age group, while the OS and PFS of the ≥75 age group were also higher than those in the 65–69 age group, although without statistical difference. These results suggest that patients in the 70–74 age group had the highest survival benefit. Given the small sample size and the nature of the retrospective study, the relationship between age and survival needs further study. Collecting tumor tissues from patients of different age groups for further pathological and biomarker detection may be helpful, although it was not performed in the present study.

In the present study, the most common AEs were anemia (40.8%), leukopenia (36.9%), gastrointestinal reaction (23.7%), fatigue (18.4%), and peripheral neuritis (17.1%), which were all manageable. The incidence of grade 3 AEs was 14.5%. No grade 4 AEs or deaths were found. This was generally comparable to the trials of nab‐paclitaxel in elderly patients with NSCLC.^11^, ^12^, ^32^, ^33^

This study had other limitations. It was a retrospective study with all the inherent biases and cons. Furthermore, it was a single‐center study with a small sample size. In addition, the data of all patients were not available for the determination of treatment response. The selection criteria also limited the generalizability of the results. Finally, 15 patients had *EGFR* mutations and might have received tyrosine kinase inhibitors, but OS was defined as the time from the initiation of nab‐paclitaxel to death. Therefore, the survival time before nab‐paclitaxel was not included in the survival analysis. The eventual benefit of EGFR‐tyrosine kinase inhibitors was probably diluted and could not be observed. Additional studies, particularly clinical trials, are still needed to examine the best treatments in elderly patients with NSCLC.

Nab‐paclitaxel had a good clinical response profile in Chinese elderly patients with stage IIIB–IV NSCLC. The AEs were tolerable and manageable. Patients of ≥70 years of age might benefit more from nab‐paclitaxel compared with younger patients. The results suggest a novel chemotherapy regimen that could benefit elderly patients with NSCLC, who are often not included in clinical trials. Prospective clinical trials are needed to confirm the results.

## Disclosure

The authors declare no competing interests.
